# A Growth-Curve Analysis of the Effects of Future-Thought Priming on Insight and Analytical Problem-Solving

**DOI:** 10.3389/fpsyg.2018.01311

**Published:** 2018-07-30

**Authors:** Monica Truelove-Hill, Brian A. Erickson, Julia Anderson, Mary Kossoyan, John Kounios

**Affiliations:** Department of Psychology, Drexel University, Philadelphia, PA, United States

**Keywords:** creativity, problem solving, temporal construal, growth curve analysis, insight

## Abstract

Research based on construal level theory (CLT) suggests that thinking about the distant future can prime people to solve problems by insight (i.e., an “aha” moment) while thinking about the near future can prime them to solve problems analytically. In this study, we used a novel method to elucidate the time-course of temporal priming effects on creative problem solving. Specifically, we used growth-curve analysis (GCA) to examine the time-course of priming while participants solved a series of brief verbal problems. Participants were tested in two counterbalanced sessions in a within-subject experimental design; one session featured near-future priming and the other featured far-future priming. Our results suggest high-level construal may temporarily enhance analytical thinking; far-future priming caused transient facilitation of analytical solving while near-future priming induced weaker, transient facilitation of insightful solving. However, this effect is short-lived; priming produced no significant differences in the total number of insights and analytical solutions. Given the fleeting nature of these effects, future studies should consider implementing methodology that allows for aspects of the time-course of priming effects to be examined. A method such as GCA may reveal mild effects that would be otherwise missed using other types of analyses.

## Introduction

Construal level theory (CLT) proposes that psychological distance from the self determines the way that one represents an object or event through mental construal (Trope and Liberman, 2003, 2010). High-level construals encompass the abstract, general features of an event or object. They omit the fine details about an object in favor of a broader representation of the object’s features (Trope and Liberman, 2010). Conversely, low-level construals include the context-dependent, concrete features of events or objects. For example, moving from ‘animal’ to ‘mammal’ to ‘canine’ to ‘dog’ represents a gradual shift from high-level to low-level construal. According to CLT, events that are psychologically distant will be represented by high-level, abstract construals, while those that are psychologically proximal will be represented by low-level concrete construals. Temporal distance reflects psychological distance in time of an event from the individual.

In line with CLT, thinking about the distant future requires more high-level construals than thinking about the near future, the latter requiring more low-level construals. In other words, individuals will form a more abstract mental representation of an event in the distant future than of an event in the near future. Because the near future is relatively proximal to the present, one has a more concrete idea of what to expect of events that occur in this time period. The distant future, on the other hand, requires more imagination—the context is unknown, and factors that are relevant to the present may change in the meantime. For example, when someone is planning a trip in the near future, there are very specific deadlines that must be met. Tickets must be booked, accommodations must be arranged, and even minor details such as the upcoming weather are known and may be incorporated in one’s decisions. If a trip is taking place in the distant future, the planning is much more abstract. General ideas such as where to go and what to do may be identified, but the concrete details cannot be considered until the trip is much closer.

Research by Liberman et al. (2002) supports this idea. In one study, participants were asked to think about completing everyday life tasks in either the distant future (1 year from the present) or the near future (1 week from the present). Participants in the distant future condition rated their ability to cope with a wide variety of everyday life tasks more similarly than those in the near future condition, suggesting less nuance in the way that distant-future tasks are conceptualized compared to near future tasks. Additionally, participants who underwent distant future priming implemented broader categories when sorting objects than those who underwent near future priming, suggesting that the more abstract mindset promoted by distant future thought can be generalized to other tasks. Other research has substantiated this idea—inducing a more abstract mindset may influence, for example, how consumers perceive advertisements (Martin et al., 2009) and how individuals deploy self-presentation strategies (Carter and Sanna, 2008). Another area which may be influenced by temporal construal priming is the method by which someone solves problems.

One of the methods people commonly use when confronted with a problem is to consciously manipulate the elements of the problem until a solution is derived. In this *analytical* approach, one works through a problem, step by step, and gradually comes to a solution. For example, one typically uses analytical problem solving when faced with an arithmetic problem. Another method by which one may solve a problem is through *insight*, commonly considered a form of creative cognition (for a discussion of the relationship between creativity and insight, see Kounios and Beeman, 2015). To solve by insight involves a sudden restructuring of the problem so that the solution is immediately clear. Unlike analytical solving (DeWall et al., 2008), insight solving is largely the result of unconscious processing (Kounios and Beeman, 2014); one’s subjective experience is that the solution came from nowhere (Schooler and Melcher, 1995). Indeed, research has shown that participants are able to rate their nearness to a solution in the case of analytical solving, but not for insight solving (Metcalfe and Wiebe, 1987). In this study, we tested whether these two problem-solving styles would be differentially affected by temporal construal priming.

Research has already shown that problem-solving style may be affected by a person’s prior internal state (see review by Kounios and Beeman, 2014). For example, neural activity immediately preceding the presentation of a problem predicts whether participants will solve that problem insightfully or analytically (Kounios et al., 2006). Subramaniam et al. (2009) showed that mood may also influence one’s brain state; in their study, a positive mood facilitated insightful solving, while an anxious mood enhanced analytical solving. Furthermore, resting-state brain activity predicts individual differences in problem-solving strategies: Participants who tend to rely more on insight exhibit different patterns of prior resting-state electroencephalogram (EEG) brain activity than those who tend to rely on analysis (Kounios et al., 2008). In sum, neuroimaging findings are consistent with the idea that mindset changes via temporal construal priming could have a significant influence on cognitive style.

A behavioral study by Förster et al. (2004) suggested that temporal construal priming influences problem-solving style. Specifically, they hypothesized that high-level construals utilized to imagine the distant future would promote insightful problem solving and that low-level construals utilized to imagine the near future would promote analytical solving. In a series of experiments, participants were asked to both imagine their life in general and imagine solving the subsequent task either in the distant future (1 year from the present day) or the near future (the next day). They reported that individuals asked to think about the distant future solved more insight problems, performed better on a creativity task, and performed worse on an analytical task. Often in creative problem-solving, one must overcome a cognitive fixation on how they assume the problem should be solved to restructure the problem in a novel manner (Smith, 1995). This fixation would be more difficult to overcome if a problem is presented in a greater level of detail, as might be expected for concrete, low-level construal. Research has supported this—when individuals were given examples on how to solve a problem, they were less likely to produce novel solutions than participants who were not provided with examples (Marsh et al., 1999). Therefore, it seems intuitive that high-level, abstract construal would benefit insight, as Förster and colleagues hypothesized. Indeed, previous research has shown that approaching a problem in a more abstract manner leads to more novel solutions than when the task is approached more concretely (Ward et al., 2004).

However, studies in which a specific mindset is primed in order to observe its effect on subsequent behavior have proven difficult to replicate (e.g., Gong and Medin, 2012; Pashler et al., 2012; Shanks et al., 2013). This report examines the consequences of mindset priming for problem-solving style. In particular, we applied a new analytic approach to investigate the time-course of the effects of future thought priming on analytical solving versus solving by insight. The present study had two main goals. The first was to test whether distant prospection benefits insight while more proximal prospection benefits analytical thinking, as suggested by Förster et al. (2004). Because of recent concerns about the replicability of social priming studies (Kahneman, 2012) we deemed it worthwhile to examine this issue.

Second, we implemented several methodological refinements to better isolate and elucidate the effects of priming. Förster et al. (2004) tasked their participants with solving both verbal and visual insight problems but did not verify whether their participants actually solved these problems with insight. Insight research has shown that just because a person has solved a so-called “insight problem” does not mean that he or she solved it with insight (Kounios and Beeman, 2014; Danek et al., 2016). We used the insight judgment procedure developed by Bowden et al. (2005) to determine which problems were solved insightfully and which were solved analytically. Instead of using classic insight problems which take participants a considerable amount of time to solve (when they are able to solve them), we used compound remote associates (CRA) problems, verbal puzzles which can be solved in less than 15 s and which have a long history of use in studying creativity and insight (Bowden and Jung-Beeman, 2003). CRAs are well-defined, convergent problems. Each CRA problem consists of 3 stimulus words that can be combined with a single solution word to form 3 individual compound words or phrases (e.g., horse, plant, over; solution = power: horsepower, power plant, overpower). Importantly, CRA problems can be solved either by insight or analysis. Based on an individual participant’s trial-by-trial reports of their solution strategy, insightful and analytical solutions can be sorted and compared. One of the major benefits of this approach is that it allows the experimenter to compare solving strategies while holding constant the type of problem.

Another benefit of using short puzzles is that it allows researchers to trace the time-course of priming effects on solving strategy. One reason that priming effects are difficult to replicate may be because these effects are too short-lived to reliably influence a subsequent task. We were able to assess this possibility by adapting growth curve analysis (GCA) to examine the time-course of temporal construal priming. GCA is a type of multilevel regression that allows for the analysis of the trajectory of time-course data (Mirman, 2014) so that one can examine change in the data over time. Using more traditional statistical methods (e.g., *t*-tests), one can compare between individual time points. However, these methods provide no information about what is happening across those time points. Using GCA, one can observe the patterns of change that occur across time points.

Growth curve analysis models are developed based on the shape of the data, fixed effects (group-level predictor variables), and random effects (variables that represent individual variability). In many cases, a linear model is a suitable reflection of time-course data. Indeed, if the priming effect persists throughout the experiment, we would expect that a linear model would best fit the data as the primed behavior would remain relatively stable. However, a linear model would not accurately identify the deterioration of priming effects over the course of an experiment. Rather, a quadratic model would successfully reveal this pattern, as one would expect an initial increase in the primed behavior, followed by a decline as the effect decays. Therefore, GCA is a useful analysis that allows for the examination of the nature of the priming effect. If these effects deteriorate over the course of a short experiment, priming researchers should take that into account during future study development. This is particularly important for those who utilize classic insight problems in their research, as these problems may require extensive solving time. Depending upon the number of problems used, the effect of priming may deteriorate before all problems have been solved.

Additionally, to maximize statistical power, we used a within-subject experimental design in which each participant was included in both a near-future and far-future thought condition (in separate counterbalanced sessions). This contrasts with the lower-power between-group design of Förster et al. (2004) and most other social priming studies.

Finally, given that one’s brain activity before a problem is presented is known to influence the strategy with which one solves the problem (Kounios and Beeman, 2014), we also measured participants’ resting-state electroencephalograms (EEG) between priming phases in order to ascertain how such priming affects ongoing brain activity.

In sum, we tested the effects of temporal-construal priming on problem-solving style (insightful versus analytic) and examined the time-course and neural correlates of the resulting effects.

## Materials and Methods

This study was carried out in accordance with the recommendations of the Drexel University IRB with written informed consent from all the subjects. All subjects gave written informed consent in accordance with the Declaration of Helsinki. The protocol was approved by the Drexel University IRB. The data are available for download at: https://figshare.com/articles/Temporal_Priming_Creative_Insight/4007745.

### Participants

Förster et al. (2004) reported large effects of temporal priming. Furthermore, based on past EEG studies with the insight judgment procedure and a within-subject design (e.g., Kounios et al., 2008), we expected that approximately 25 participants would yield good statistical power for analyses of both the behavioral and EEG data. Given expected participant exclusions due to EEG artifacts, low problem-solving accuracy, failure to follow instructions, and participant withdrawals, we recruited 38 participants.

All participants were right-handed, had no self-reported neurological disorders or psychiatric conditions, and refrained from taking substances that might affect cognition (i.e., alcohol, psychoactive medications, or recreational drugs) for 24 h prior to the experiment. We excluded 2 participants who did not produce at least 1 solution of each type (insight and analytic) because this suggested that they were responding stereotypically or were not following instructions. We also excluded 2 subjects who did not achieve an accuracy lower than 1.5 standard deviations below the sample mean (∼15% accuracy) in solving the problems, 3 due to equipment problems, and 4 who chose not to complete the study. After these exclusions, our final sample included 27 Drexel University students ages 18-30 (*M* = 22.15, *SD* = 3.28, 13 females, 13 males, 1 declined to report) who were paid $30 to participate.

### Procedure

**FIGURE 1:**
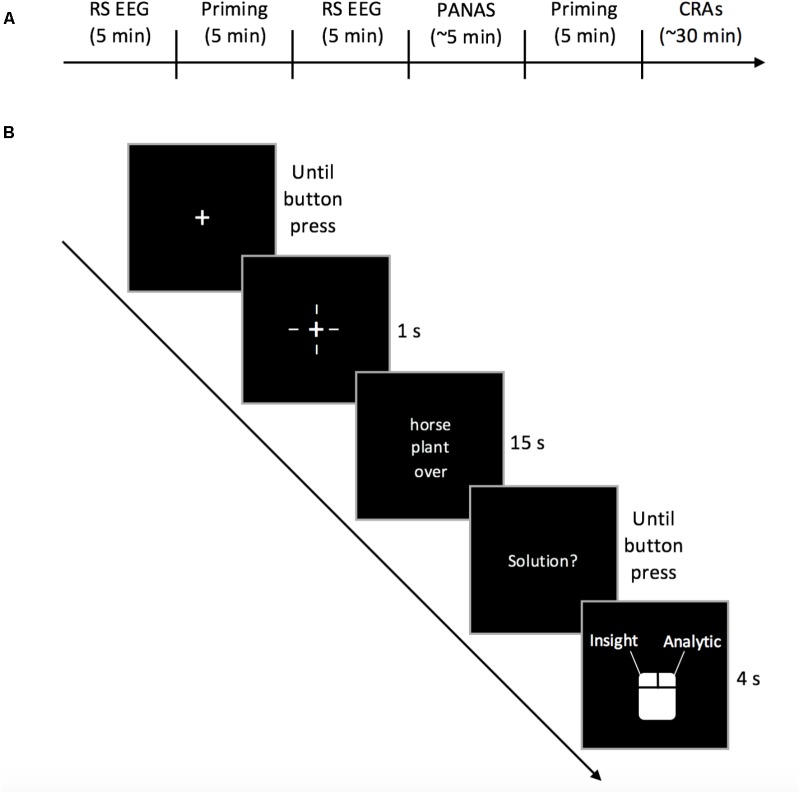
**(A)** Experimental procedure timeline and **(B)** CRA procedure.

Participants completed 2 2-h experimental sessions on different days. During the first session, participants filled out demographic and handedness questionnaires and watched an instructional video during which the experimental procedure was explained and the differences between analytical and insightful problem solving were described. We recorded 5 min of eyes-closed baseline resting-state EEG data during which participants were instructed to let their minds wander. Then, participants were presented with 1 of 4 possible priming scenarios (2 in the near condition and 2 in the far condition, as described below) and asked to write about that scenario for 5 min. After this priming, we recorded 5 min of eyes-closed resting-state EEG data. Because of the documented effects of mood on insight (Subramaniam et al., 2009), participants then completed the Positive and Negative Affect Scale (PANAS). Participants completed another priming scenario (same time-frame) for 5 min to refresh the priming after the EEG recording and PANAS. Following this, participants attempted 72 CRA problems while recording EEG. The second session used the same procedure (**Figure 1**). Participants who received far-future priming scenarios in the first session received near-future scenarios in the second session, and vice versa.

### Materials

#### Priming Scenarios

We used 4 priming scenarios, differing both in content and temporal proximity. The scenarios were restricted to the Philadelphia area to control for potential spatial-distance priming effects. The scenarios are as follows:

• “Imagine that you will be finding a place to live in Philadelphia next week. You have 5 min to write about whatever comes to mind about this.”
• “Imagine that you will be finding a new job in Philadelphia next week…”
• “Imagine that you will be finding a place to live in Philadelphia in 10 years…”
• “Imagine that you will be finding a new job in Philadelphia in 10 years…”

#### Compound Remote Associates

Participants were presented with 144 CRA problems over the course of the study. The assignment of CRA problems to sets was randomized and the sets were counterbalanced between groups. Each set of CRAs was presented to participants in a single random order. The problems were presented using e-Prime 2.0. Eight practice trials were presented before each session. Participants held a mouse in both hands with left and right thumbs placed on the corresponding buttons. A fixation cross was displayed in the center of the screen until participants initiated the presentation of a problem with a bimanual button press. Once participants initiated the problem, crosshairs appeared around the fixation cross for 1000 ms after which the problem appeared. The 3 words of each problem were displayed in a column for 15 s. If a participant was unable to reach a solution, the screen returned to the fixation cross and the trial was terminated. If a participant reached a solution, she or he indicated this with a bimanual button-press. Then, a prompt appeared on the screen, participants verbalized their solution, and the experimenter recorded solution accuracy. Participants were then prompted to press a button to indicate whether they had solved the problem insightfully (i.e., resulting from an “aha” moment in which the solution suddenly intrudes on ongoing thought) or analytically (i.e., in which the solution resulted from deliberate, conscious manipulation of the elements of the problem, as in hypothesis testing; Bowden et al., 2005). If participants were unable to come to a conclusion as to how the problem was solved, they refrained from pressing anything, and the program continued after 4 s.

### EEG Recording and Data Processing

Eighty-four channel electroencephalographic data were recorded with tin electrodes embedded in a nylon cap (Electro-Cap International, Eaton, OH, United States) using the MANSCAN EEG recording system (SAM Technology, Inc., San Francisco, CA, United States) and extended 10–20 system locations referenced to digitally linked mastoid electrodes. Data were preprocessed using the EEGLAB toolbox in Matlab 7.14 (Mathworks, Inc., Natick, MA, United States). Bad channels were removed by visual inspection. Data were segmented and filtered using a 1-Hz high-pass and 55-Hz low-pass FIR filter. Movement artifacts were removed using an amplitude threshold ranging from -300 to 300 μV (Hoffman and Falkenstein, 2008). ICA weights were calculated using EEGLAB’s FASTICA algorithm and submitted to the ADJUST artifacting tool (Mognon et al., 2011). Previously removed channels were replaced by interpolation. Analyses were conducted in SPM 12’s EEG toolbox (Litvak et al., 2011). Fast Fourier transforms (FFT) were calculated from 2 to 55 Hz in frequency steps of 2 Hz (Hamming windowed), robust averaged, and log transformed within session, then transformed into 3D Scalp × Frequency images. Tests were performed with a *p* < 0.001 cluster-correction threshold.

### Behavioral Data Analysis

Growth-curve analysis (Mirman, 2014) was used to analyze change over time in the relative accumulation of solutions over the course of the 72 CRA problems presented during each session. GCA offered information both about the influence of priming on solution type and the time course of this influence. All analyses were undertaken with R version 3.1.1 using the lme4 package (version 1.1-7).

#### Solution Difference (Insight – Analytical Solutions)

The time-course of changes in solving style (insight versus analysis) was modeled with second-order orthogonal polynomials using fixed effects of priming on all time terms (in all analyses in this report, this refers to the intercept, linear, and quadratic terms) and with participant and participant-by-condition (near versus far priming) random effects on all time terms. In this analysis, the intercept term refers to the average solution difference score, the linear term refers to the change in the solution difference score over time, and the quadratic term captures the curvature of the data—specifically, the increase and then subsequent decrease of the solution difference score over time, or vice versa. The far-priming condition was treated as baseline with parameters being estimated for the near-priming condition. Parameter-specific *p*-values were estimated using the normal approximation.

#### Solution Accumulation

The overall time-course for each condition (near versus far priming) was modeled with second-order orthogonal polynomials using fixed effects of solution type on all time terms and with participant and participant-by-solution type (analytical versus insight solution) random effects on all time terms. In this analysis, the intercept term refers to the average number of each solution type, the linear term captures the solution accumulation rate, and the quadratic term reflects the change the rate of solution accumulation over the course of the experiment. Insight solutions were treated as baseline with parameters being estimated for analytical solutions. Parameter specific *p*-values were estimated using the normal approximation.

## Results

### Mean Performance

In the far-future priming condition, participants reported an average of 11.30 (*SD* = 6.03) correct insight solutions and 12.26 (*SD* = 7.34) correct analytical solutions. They solved 8.44 (*SD* = 11.41) problems incorrectly, and timed out in 39.44 (*SD* = 11.61) trials. In the near-future priming condition, participants reported an average of 10.78 (*SD* = 5.44) correct insight solutions and 10.81 (*SD* = 5.76) analytical solutions. They solved an average of 10.30 (*SD* = 14.31) problems incorrectly, and timed out in 38.85 (*SD* = 14.34) trials. Neither the positive affect (*t* = -1.00, *p* = 0.329) nor the negative affect (*t* = 0.486, *p* = 0.632) PANAS scores significantly differed between conditions (see **Table 1**).

**Table 1:** Positive and negative affect scores by condition.

	PAS		NAS	
	Mean	*SD*	Mean	*SD*
Near-future condition	34.96	5.56	21.79	7.95
Far-future condition	33.96	5.63	22.46	6.58


There were no significant differences between priming conditions in terms of total correct solutions (*p* = 0.106), total incorrect solutions (*p* = 0.429), and total timeouts (*p* = 0.897). Data for all of the following models can be found in **Table 2**.

**Table 2:** Model fit results for each analysis.

	Solution difference (I – A)			Near-future priming			Far-future priming		
	LL	χ^2^	*p*	LL	χ^2^	*p*	LL	χ^2^	*p*
Base model	-5933.3	–	–	-3945.2	–	–	-4369.6	–	–
Intercept	-5931.9	2.75	0.097	-3943.9	2.54	0.111	-4368.6	1.85	0.174
Linear	-5872.6	118.46	<0.001^∗^	-3943.9	0.08	0.771	-4368.6	0.16	0.687
Quadratic	-5866.2	12.75	<0.001^∗^	-3940.3	7.14	0.008^∗^	-4364.5	8.1	0.004^∗^


*^∗^Significant improvement in model fit.*

### Solution Difference-Scores

**FIGURE 2:**
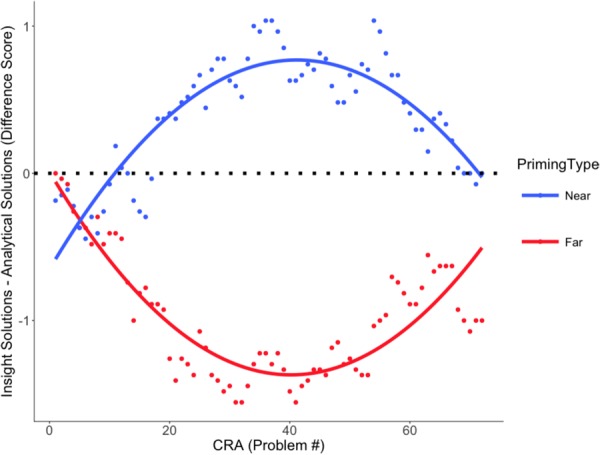
Solution difference score by priming type. Model fit of the solution difference score (insight solutions – analytical solutions) by priming type (near-future versus far-future) over the series of CRA problems.

The effect of priming significantly improved model fit on the quadratic term, χ^2^ = 12.75, *p* < 0.001, indicating that a curvilinear model best fits the data. Solution pattern and consistency differed significantly between conditions over the course of the experiment, as reflected by differences in the steepness of the quadratic curvature between the near- and far-future priming conditions. Specifically, participants in the far-future priming condition produced consistently more analytical solutions in the initial stages of the experiment, *Estimate* = 2.80, *SE* = 1.12, *p* = 0.013. Conversely, significance in the opposite direction in the near-future priming condition indicates that participants utilized more insightful solving immediately after priming, *Estimate* = -5.54, *SE* = 1.51, *p* < 0.001. This difference grew smaller as the experiment progressed (**Figure 2**).

### Near-Future Priming Condition

The effect of solution type significantly improved model fit on the quadratic term, χ^2^ = 7.14, *p* = 0.008, indicating a curvilinear model as the best fit of the data. Solution type did not significantly affect the intercept or the linear terms, *p* = 0.599, indicating that there was no significant difference in solution type in the near-priming condition; overall, participants tended to apply analytical and insightful methods about equally often. However, the effect of solution type on the quadratic term reflects differences in the steepness of quadratic curvature between the two conditions. This can be related to solution-type accumulation over time. Specifically, with near-future priming, insights initially accumulated somewhat more rapidly than analytical solutions, *Estimate* = -2.47, *SE* = 0.73, *p* = 0.001. However, the curvature of analytical solutions was also significant, but in the opposite direction, *Estimate* = 2.75, *SE* = 0.99, *p* = 0.006, which suggests that they were mildly suppressed by near-future priming.

**FIGURE 3:**
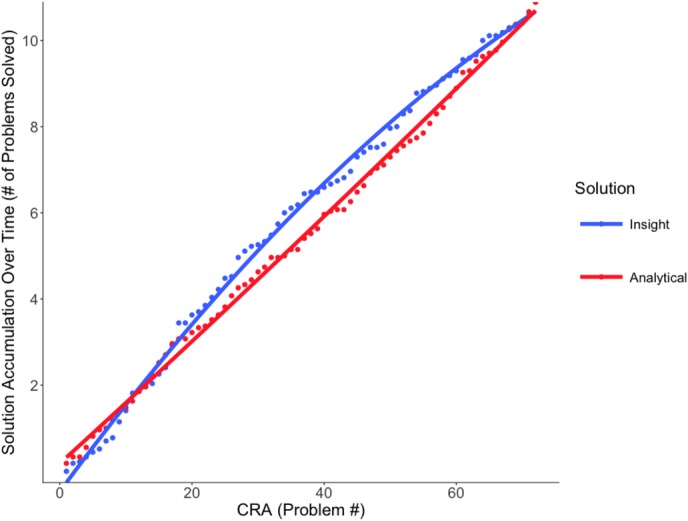
Near-future priming solution accumulation. Model fit of the accumulated solutions (insight versus analytical) over the series of CRA problems in the near-future priming condition. Although participants used roughly the same number of each solution type, insightful solutions initially accumulated somewhat more rapidly than analytical solutions.

Although participants applied roughly equal numbers of insightful and analytical solving methods over the course of the experiment, the rate of accumulation of each solution type differed (**Figure 3**). Insightful solutions accumulated slightly more rapidly than analytical solutions in the initial portion of the experiment.

### Far-Future Priming Condition

**FIGURE 4:**
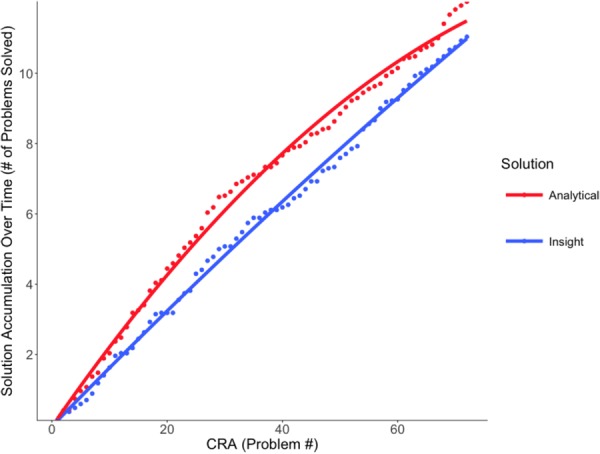
Far-future priming solution accumulation. Model fit of the accumulated solutions (insight versus analytical) over the series of CRA problems in the far-future priming condition. Although participants used similar numbers of each solution type, analytical solutions initially accumulated more rapidly than insightful solutions.

The effect of solution type significantly improved model fit on the quadratic term, χ^2^ = 8.10, *p* = 0.004, indicating a curvilinear model as the best fit of the data (**Figure 4**). As in the near-priming condition, there was no significant difference in solution type, *p* = 0.306, but, rather, there was a significant difference in the rate of solution accumulation over time, as indicated by the steepness of the curvature in the analytical condition, *Estimate* = -2.80, *SE* = 0.95, *p* = 0.003. Specifically, analytical solutions initially accumulated more rapidly than insights.

Similar to the near-future priming condition, participants utilized relatively equal numbers of insightful and analytical solutions over the course of the experiment. However, the rate of accumulation differed. In this condition, analytical solutions accumulated more rapidly than insightful solutions in the initial portion of the experiment.

### Resting-State EEG Data

**FIGURE 5:**
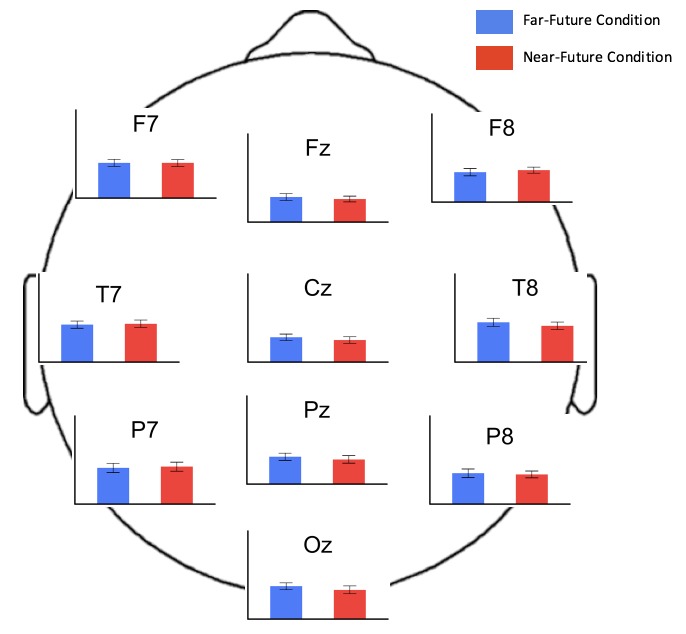
Mean EEG beta-frequency power values in log (μV2) for selected electrode sites by priming condition. All bar charts are scaled 0 to -10 in units of log(μV2), with negative plotted up and error bars reflecting the standard error.

The resting-state EEGs were subjected to frequency-domain analyses. To test for priming differences across all frequency bands (2–50 Hz), a flexible factorial model was created with the factors *order* (of priming condition) and *priming-condition* (near- versus far-future conditions). The first contrast tested the main effect of priming-condition in an *F*-test. No clusters survived at a cluster-forming threshold of *p* < 0.001. Because in-preparation analysis of other resting state data that we have collected shows that differences in resting-state beta-band oscillations are the strongest predictor of subsequent problem-solving strategy, we performed a focused analysis of priming condition constrained to the beta band (13–30 Hz). Again, no clusters survived at a cluster-forming threshold of *p* < 0.001. In sum, these analyses revealed no significant brain-activity differences between the near-future and far-future priming conditions after 5 min of priming (**Figure 5**). Means of the logged beta EEG power values for selected representative electrodes are shown in **Table 3**.

**Table 3:** EEG beta power values for selected electrodes log(μV^2^).

	Near-future		Far-future	
Electrode	Mean	*SD*	Mean	*SD*
Fz	-2.50	1.85	-2.46	2.43
F7	-3.98	1.97	-4.00	2.02
F8	-3.62	1.73	-3.40	2.12
Cz	-2.26	2.17	-2.20	2.59
T7	-4.79	2.32	-4.49	2.69
T8	-4.05	2.26	-4.51	2.34
Pz	-2.66	2.36	-2.62	2.69
P7	-4.19	2.72	-3.95	2.89
P8	-3.61	2.33	-3.62	3.05
Oz	-2.85	2.86	-2.73	3.27


## Discussion

Research by Förster et al. (2004) indicates that thinking about the distant future promotes both creative processes (such as insight) and creative outputs and suppresses analytical reasoning. Our data contradict this. Distant-future thought primed analytical problem solving while near-future thinking primed insightful solving. Moreover, the shapes of the fitted curves illustrate a deterioration of these priming effects over approximately 30 min (the time course of the stimulus presentation procedure). The priming effect was more pronounced in the far-future priming condition than in the near-future condition. This was not unexpected because the near future is similar to the present. Far-future thought would plausibly induce a greater change in mind-set and a more pronounced priming effect because the far future is comparatively dissimilar to the present.

One possible explanation for the difference between our findings and Förster et al.’s (2004) is that future-thought priming effects may be highly dependent on the specific content of the priming scenarios. For example, our scenarios may have prompted more concrete construals, regardless of priming condition, than those used in the Förster et al. (2004) study. Thinking about detail-oriented tasks such as finding a place to live or finding a job may produce an inherently more low-level construal than thinking about life in general. However, if this were the case, then we might expect predominantly analytical solutions in both priming conditions. This did not occur – near-future priming gave a small temporary boost to insightful solving. Another hypothesis is that the priming scenarios could have induced mood changes strong enough to override temporal-construal priming (Subramaniam et al., 2009). However, the absence of any significant priming effects on the PANAS mood questionnaire results weighs against this hypothesis. Finally, it is possible that the tasks that Förster et al. (2004) used did not tap creativity or insight and that their participants were using analytic thought to accomplish them. Because participants may solve so-called classic insight problems by using analytical methods (Danek et al., 2016), the present study used a method that revealed on a trial-by-trial basis the type of processing that each participant used to solve each CRA problem.

One potential explanation for our findings is that high-level construal, such as thinking about the distant future, may engage executive processes involved in working memory maintenance and inhibition of prepotent long-term memory representations more than low-level construal. Indeed, several studies indicate that imagining a future event draws heavily on working memory and other executive processes required for analytical problem solving (e.g., D’Argembeau et al., 2010; Zavagnin et al., 2016). It is expected that far-future priming would draw more heavily upon these processes than near-future priming because an event in the near future is very similar to an event in the present. Specifically, imagining that you are looking for a job next week is not significantly different than imagining that you are currently looking for a job. The only details that must be retained in working memory are the few slight deviations from one’s current situation; namely, that one has to find a job. In contrast, one is likely to assume that things will be quite different 10 years from the present. One may assume that they are married, possibly with children, and may have other family responsibilities or interests. They will likely expect to have different career options than they presently have. Thus, when imagining the distant future, one has to maintain in working memory all of these new features, while inhibiting some features of the present that conflict with those being imagined. In essence, imagining the distant future is likely a more computationally complex simulation than imagining the near-future. These findings, together with research indicating that enhanced analytical problem solving depends on working memory capacity more than insightful problem solving (Fleck, 2008; Wiley and Jarosz, 2012; DeCaro, 2016), lend credence to the idea that thinking about the distant future primes analytical thinking by activating these executive processes.

Interestingly, the temporary facilitation of analytical problem-solving in the distant-future condition did not produce a significant change in the total number of analytical solutions compared to insights. This suggests that not only does the priming’s facilitating effect deteriorate, but analytical solving may actually be suppressed for a short time, as in a rebound effect. Because analytical problem-solving requires deliberate, focused attention (Kahneman, 2011) and because executive processes are susceptible to resource depletion (e.g., van der Linden et al., 2003; Persson et al., 2007), it is plausible that a rebound effect may occur due to cognitive fatigue from sustained analytical thought. This rebound effect is not as robust in the near-future priming condition, which may be in keeping with the idea that insightful problem-solving is largely unconscious (Fleck, 2008), and would plausibly induce less cognitive fatigue. However, it may also be less robust because the effect of near-priming is weaker in general.

Regarding the temporary nature of the priming effect, there are two important implications. The relative brevity of such effects may be responsible for some previous failures to replicate social priming effects if the test phases of those experiments were either too long or too delayed after a weak priming phase. Indeed, had we examined behavioral priming effects averaged over the session rather than analyzing the time-courses of these priming effects, we could have missed them altogether. This is consistent with other recent research that suggests that priming effects of future thought may not be as robust as previously suggested (Stins et al., 2016). Thus, the dynamic properties of priming should be taken into consideration in future studies.

Furthermore, though we observed temporary priming effects on behavior after participants received two 5-min priming sessions, the first 5-min priming phase was insufficient to cause any detectable changes in resting-state brain activity, the likely mediator of priming effects (Kounios et al., 2008). This is likely because the priming duration was too brief. Although behavioral effects could be observed after 10 min of priming, these were short-lived. This indicates that the effect of priming on problem-solving is relatively weak. Some prior priming studies have used short periods of even less-immersive priming, thus decreasing the likelihood of obtaining even subtle effects.

To summarize, growth-curve analysis showed that high-level construals engaged by distant-future thought transiently primed analytical solving while low-level construals engaged by near-future thought transiently primed insightful solving. Further research should investigate whether the direction, duration, and intensity of such priming effects are determined by specific features of the priming scenarios and whether other types of priming are similarly fleeting.

## Author Contributions

JK devised the conceptual idea for the study. All authors contributed to study design. JA and MK collected pilot data. MT-H and BE collected the data and performed the data processing and analysis. MT-H wrote the manuscript with support from JK.

## Conflict of Interest Statement

The authors declare that the research was conducted in the absence of any commercial or financial relationships that could be construed as a potential conflict of interest.
